# Transjugular Intrahepatic Portosystemic Shunt in Nonmalignant Noncirrhotic Portal Vein Thrombosis and Portosinusoidal Vascular Disorder

**DOI:** 10.3390/jcm13051412

**Published:** 2024-02-29

**Authors:** Sarah Shalaby, Roberto Miraglia, Marco Senzolo

**Affiliations:** 1 Department of Surgery, Oncology and Gastroenterology, Padua University Hospital, 35128 Padua, Italy; marcosenzolo@hotmail.com; 2 Radiology Service, IRCCS-ISMETT (Mediterranean Institute for Transplantation and Advanced Specialized Therapies), 90127 Palermo, Italy; rmiraglia@ismett.edu

**Keywords:** transjugular portosystemic shunt, acute portal vein thrombosis, cavernoma, portosinusoidal vascular disorder, non-cirrhotic portal hypertension

## Abstract

Transjugular intrahepatic portosystemic shunt (TIPS) emerges as a key treatment for portal hypertension (PH) complications. While international guidelines provide clear indications for its use in cirrhosis, empirical knowledge is notably scarcer in non-cirrhotic PH, particularly in nonmalignant noncirrhotic portal vein thrombosis (NNPVT) and in patients with portosinusoidal vascular disorder (PSVD). Patients afflicted by these rare diseases exhibit distinct clinical profiles compared to their cirrhotic counterparts, often characterized by a younger age, predominantly preserved hepatic functionality even in cases of severe PH, and a higher propensity for extensive splanchnic thrombosis, which intricately complicates TIPS placement, posing unique challenges for its creation. The objective of this review is to synthesize existing literature on the effectiveness, safety, specific indications, and clinical outcomes of TIPS in adult patients with NNPVT or PSVD, focusing also on the technical challenges of TIPS insertion in the presence of portal cavernoma.

## 1. Introduction

Transjugular intrahepatic portosystemic shunt (TIPS) emerges as a key treatment for portal hypertension (PH) complications in patients with cirrhosis, serving either as a definitive solution or a bridge to transplantation [1]. While the indications and timing for the application of TIPS in patients with cirrhosis are well defined by guidelines and consensus manuscripts [2,3,4,5], there is a significant gap of knowledge in the context of non-cirrhotic PH. The majority of evidence in this context regards the use of TIPS in the stepwise treatment of Budd-Chiari syndrome [6,7], but guidance on using TIPS for nonmalignant noncirrhotic portal vein thrombosis remains sparse, particularly in complex cases. Moreover, limited data exists on TIPS’s impact on portosinusoidal vascular disorder (PSVD), given the rarity of this clinical condition. Patients with non-cirrhotic PH often differ from those with cirrhosis; they tend to be younger, have generally preserved liver function even in cases of severe PH, and experience splanchnic thrombosis much more often than cirrhotic patients, which can be extensive and can complicate the creation of a TIPS. The aim of this review is to evaluate current literature regarding the effectiveness, timing, indications, and clinical outcomes of the use of TIPS in adult patients with nonmalignant noncirrhotic portal vein thrombosis and PSVD, with an emphasis on the technical aspects of TIPS placement in cases of portal cavernoma.

### 1.1. Nonmalignant Noncirrhotic Portal Vein Thrombosis

Nonmalignant noncirrhotic portal vein thrombosis (NNPVT) refers to a thrombus formation in the portal vein, with or without concomitant involvement of the superior mesenteric vein (SMV) and/or splenic vein (SV). This condition is uncommon, particularly when chronic liver disease is absent, with a prevalence of only 1.0% in the general population [8]. In contrast to the patient with cirrhosis, a detailed thrombophilia workup is warranted in most patients without cirrhosis who develop a PVT, as in about 70% of NNPVT cases, identifiable prothrombotic factors are present, with systemic factors involved in 60% and local factors in 20–40%. Myeloproliferative neoplasms are a leading cause, although their diagnosis may be complicated by portal hypertension; thus, a close collaboration with a hematologist and a bone marrow biopsy are often necessary for confirmation. Hereditary thrombophilic factors, hormonal factors and local conditions may also play roles in NNPVT risk, emphasizing the need for a thorough evaluation [9]. Despite comprehensive investigations, 30% of cases remain idiopathic.

Advances in radiology and disease understanding have led to earlier detection of NNPVT in its acute (or “recent,” as recently proposed) phase rather than in the chronic stage. Radiological indicators for acute PVT include iso- or hypoechoic material visible on Doppler ultrasound, hyperdense material on pre-contrast computed tomography (CT) scans, and a lack of significant portoportal or portosystemic collateral. Chronic PVT refers to a thrombus persisting for more than 6 months, a duration supported by studies showing that recanalization rarely occurs after this period and that cavernous transformation (a network of twisted vessels replacing the normal portal vein) often develops despite anticoagulation. Thus, radiologic features include cavernoma formation, portosystemic collaterals, splenomegaly, calcium deposits in the vessel walls, tortuous vessel paths and reduced diameter [9]. However, a clear distinction between acute and chronic PVT in the absence of previous abdominal imaging is still challenging, as symptoms are not always present during the acute onset of PVT and a cavernoma can form as early as 1 week after PVT occurrence.

Currently, several classifications are available combining the anatomical and functional characteristics of the NNPVT that can be more or less suitable depending on the different scenarios [9]. Nevertheless, in the absence of a globally accepted unique classification, an accurate description of the extension of the thrombus and degree of occlusion of each splanchnic venous segment is currently recommended [9]. NNPVT symptoms can vary based on the timing of onset (acute vs. chronic) and the extent of the thrombus. In approximately one-third of cases, the onset of NNPVT may be asymptomatic and diagnosed accidentally during abdominal imaging performed for unrelated reasons. On the other hand, advancements in non-invasive diagnostic methods have changed the clinical history of patients with symptomatic NNPVT, making abdominal pain the most common symptom at initial diagnosis, while the incidence of gastrointestinal bleeding as the first manifestation has decreased (2.4 vs. 12.7/100/year) [10]. Indeed, the most severe consequences of NNPVT include intestinal ischemia when the SMV is involved, which can lead to intestinal necrosis, the development of pre-hepatic PH, and the compression of nearby structures by collateral vessels that develop in the attempt to bypass the occlusion (cavernoma) [11,12]. With regard to treatment, anticoagulation is recommended as a first-line approach, in addition to identifying any associated potentially treatable conditions by testing the patient for all known causes of thrombophilia [9,13]. Interventional radiology approaches are currently recommended in cases of refractory complications despite adequate medical treatment or when anticoagulation treatment is contraindicated [9,13]. Nevertheless, the actual benefits and timings for TIPS creation are still much debated due to the lack of randomized controlled trials. Indeed, most of the evidence derives from case reports, case series, and uncontrolled retrospective cohort studies.

#### 1.1.1. TIPS for Acute Nonmalignant Noncirrhotic NNPVT

Although optimal treatment of NNPVT remains a matter of debate, anticoagulation is regarded as the standard therapy for acute NNPVT, with the aim of restoring blood flow and preventing immediate and late complications. Anticoagulant treatment succeeds in giving partial recanalization in about 40% of cases [14,15,16,17], with higher rates if therapy is started promptly after the occurrence of acute thrombosis. The presence of ascites, the extension of thrombosis to the splenic vein, the association with prothrombotic factors and delayed initiation of anticoagulation have been described as predictors of failure to achieve recanalization despite anticoagulation [15,17,18]. In the case of extension of thrombosis to the SMV with the onset of intestinal ischemia, the timing of handling is guided by the symptoms and evolution of the ischemia rather than by imaging monitoring of recanalization. In this context, studies available in the literature report the use of TIPS mainly for the purpose of creating an access site to the splanchnic circulation to allow local mechanical and/or fibrinolytic treatment in the case of persistent intestinal ischemia regardless of adequate anticoagulation. Due to the lack of data, strong recommendations for treatment algorithms in this scenario have not yet been established. In fact, only a few studies using different approaches have currently been published and are not yet validated. The most consistent cohort has been reported by Naymagon and colleagues, who conducted a retrospective analysis of 330 patients with acute NNPVT, monitored over an average period of 41.6 months. Their findings highlight a significant success rate in achieving portal recanalization using anticoagulant monotherapy, particularly with direct oral anticoagulants. This suggests that such an approach is advisable initially for non-critical patients. However, it is important to note that none of the patients in this cohort presented with intestinal ischemia, indicating an exclusion of cases with rapid and severe clinical escalation. Additionally, the study found that 27% of patients treated with anticoagulants developed portal cavernomatosis; among these, 23% developed refractory PH and eventually needed a TIPS. Unfortunately, the authors did not provide information on this subgroup’s outcomes after TIPS creation [19]. Klinger and colleagues reported the results of 17 patients with acute NNPVT (10 with intestinal ischemia) followed-up for a median of 28.6 months who underwent transjugular local fibrinolysis and/or mechanical thrombectomy. Of these, in only eight patients who displayed hemodynamically significant residual intrahepatic thrombosis post-thrombolysis (defined by the authors as a residual portosystemic pressure gradient > 12 mmHg), a TIPS was created after local treatment using a 10 mm ePTFE-covered stent. Following this approach, there was one case of recurrent intestinal ischemia, and two patients (11.8%) required segmental intestinal resection (no detail is available on the size of the resected segment and related outcome). For those in the TIPS group, primary portal vein patency rates at 6, 12, and 24 months were 87.5%, 62.5%, and 62.5%, respectively, compared to 88.9% in patients without TIPS. The secondary portal vein patency rates at these intervals were 87.5%, mirroring the 88.9% in non-TIPS patients. The recurrence of NNPVT after initial successful recanalization was observed in 83.3% and 10% of the patients, respectively. Additionally, the study identified the JAK2 mutation as an independent predictor of NNPVT recurrence or TIPS thrombosis. Notably, no deaths or PH complications were reported during the follow-up, though data on complications related to TIPS creation were not provided [20]. Finally, Benmassaoud and colleagues developed a protocol based on the initiation of low-dose systemic thrombolysis for patients who continue to experience abdominal pain after an anticoagulation trial. The proposed algorithm escalates to the creation of a TIPS to perform local thrombolysis in case of no radiological and/or clinical improvement within 48–72 h. This approach was applied to 22 patients who did not respond to initial anticoagulation, 77% of whom had a complete occlusion of the entire porto-mesenteric axis. Following the systemic thrombolysis, 14 patients (63%) required a switch to TIPS placement, which was successfully performed in 11/14 patients. All patients underwent local thrombolysis through the TIPS, except for one for whom it was deemed unnecessary after shunt creation. Symptom resolution was achieved in 91% of patients, and recanalization was successful in 86%. Notably, only one patient required resection due to intestinal necrosis. Additionally, there were no reported deaths or major complications from systemic thrombolysis, underscoring the protocol’s safety and effectiveness [21]. While these results appear promising, most patients eventually needed to step up to local thrombolysis, questioning the real value of the intermediate step of systemic thrombolysis. Additionally, the treatment was started significantly later after symptom onset [18.7 ± 11.2 days], excluding patients who quickly developed intestinal necrosis, which represents a population of interest in this context. Based on this limited evidence, American guidelines currently recommend portal vein thrombectomy/thrombolysis using a transjugular approach with or without small-caliber TIPS in patients who fail or have a poor response to initial anticoagulation (timings not defined) [2]. Hence, there is a significant need for a comprehensive and well-designed prospective study in this area.

#### 1.1.2. TIPS for Chronic Nonmalignant Noncirrhotic NNPVT

In the case of chronic NNPVT, the recommendations on the management of anticoagulant treatment are less robust. Indeed, the absence of controlled prospective studies examining the risk-benefit profile of long-term anticoagulation in preventing re-thrombosis in chronic portal NNPVT remains a notable gap. While retrospective studies hint at its effectiveness in reducing new thrombotic episodes [14,22,23,24] and potentially enhancing survival without an increase in the risk of bleeding [22,25,26,27,28], there are also conflicting reports [16,29]. Furthermore, these studies have not differentiated outcomes based on the presence or absence of underlying prothrombotic factors in patients. Regarding the use of TIPS in this context (combined or not with other intravascular techniques), it is generally considered for the treatment of refractory complications of PH or portal cholangiopathy despite adequate anticoagulation and, less commonly, for intractable abdominal pain, primarily when the thrombus extends to the SMV. A significant ongoing debate against the use of TIPS in this setting revolves around the necessity of maintaining an intrahepatic shunt in patients without liver disease, particularly after effective recanalization of the portal system. Indeed, percutaneous portal vein recanalization (PVR), without TIPS creation, can also be considered in patients with NNPTV, without chronic liver diseases, with low liver stiffness and with patent intrahepatic portal branches [30,31]. It is important to note, however, that a more extensive intrahepatic spread of thrombosis correlates with an increased likelihood of PVR failure, often resulting in early stent thrombosis due to inadequate blood outflow. Consequently, PVR alone is not advisable for these patients and should be considered a candidate for TIPS creation. Moreover, a subset of these patients may present with concomitant pre-sinusoidal PH associated with NNPVT (i.e., PSVD), and at present, liver biopsy does not provide a definitive means of differentiating changes caused by chronic thrombosis from those due to PSVD [32]. This complexity adds to the debate and challenges of managing chronic NNPVT with TIPS. Another advantage of the use of small-caliber TIPS in the context of NNPVT is that it can secure easy re-access to the portal system should endovascular reintervention in the portal system be necessary, particularly in cases of extended thrombosis. On the other hand, the potential complications of a TIPS should be taken into account in the risk-benefit balance, making clinical decisions difficult in the absence of evidence-based support. In an attempt to facilitate this decision-making process, specific factors have been identified to help identify patients who are at high risk of re-thrombosis and therefore might benefit from more aggressive NNPVT treatment, including TIPS creation and maintenance of anticoagulation in the long term after PVR with or without TIPS. Key factors to consider are an underlying prothrombotic disorder, a previous history of thrombotic events in other anatomical sites, and the simultaneous occurrence of intestinal infarction. Moreover, in cases where no specific prothrombotic conditions are found, elevated levels of Factor VIII (above 150%) or D-Dimer (exceeding 500 ng/mL) have also been linked to a higher risk of re-thrombosis [13,33]. 

Another factor to be considered in this context is the technical difficulty of creating a TIPS. Indeed, the factors that have challenged the technical feasibility of creating a TIPS in patients with chronic NNPVT are the presence of portal cavernoma, the complete obstruction of the main portal vein, the absence of visible intrahepatic portal vein branches and the extension of thrombosis to the SMV [34,35,36,37,38]. Nevertheless, recent advances in interventional techniques have certainly improved the technical success rate. The employment of real-time ultrasound guidance during TIPS creation proves to be exceptionally beneficial, especially for patients with complete NNPVT or portal cavernoma. This technique facilitates the accurate targeting of the portal venous system. It allows for precise puncturing of intrahepatic portal branches that are thrombosed in cases where the thrombosis extends into these branches. Additionally, in patients with portal cavernoma, it enables the selective puncturing of the intrahepatic portal branch that communicates with the remnant of the portal vein, enhancing the effectiveness and safety of the TIPS creation procedure [39]. The combined transjugular/transhepatic, transjugular/transplenic or transjugular/transmesenteric approaches have been reported for TIPS creation in NNPVT patients after conventional transjugular approach failure or as a first-line approach [36,39,40,41,42,43]. On note, due to the paucity of data present in the literature, no clear recommendation can be given about the use of a transhepatic, transplenic or transmesenteric approach in such cases. Table 1 summarizes the cases described in the literature reporting the creation of a TIPS for chronic NNPVT in the presence of portal cavernoma (known to be the cases with the greatest technical difficulties) [44,45,46,47,48,49,50].

**Figure 1:**
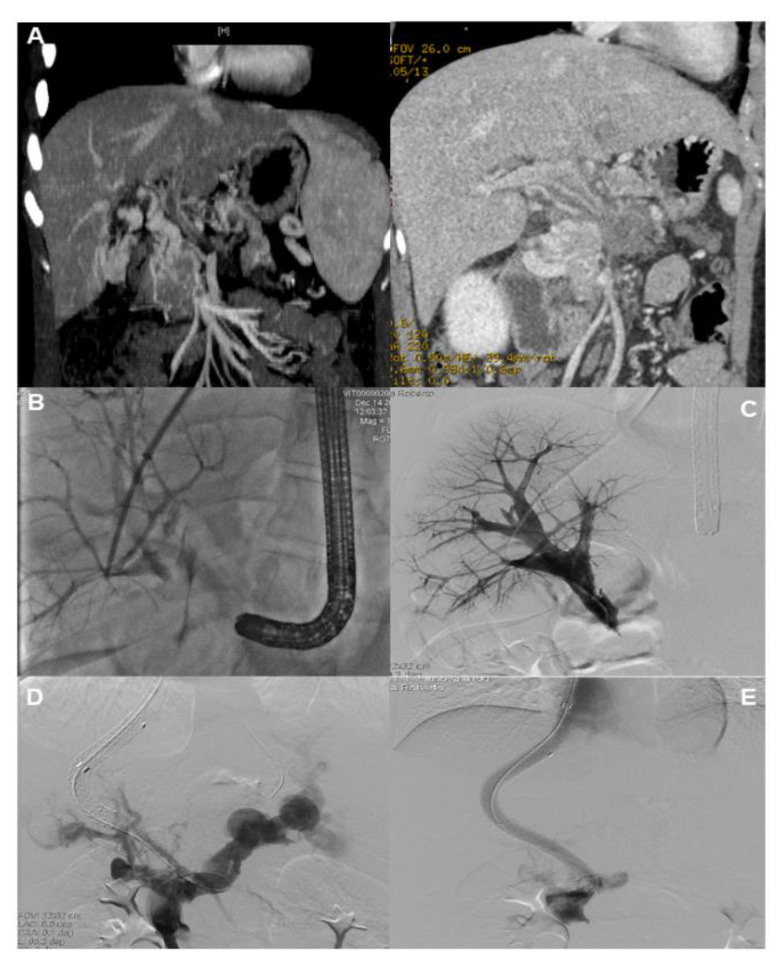
Example of a patient with noncirrhotic nonmalignant portal vein cavernoma secondary to umbilical vein catheterization in the neo-natal period. The patient was admitted for uncontrolled upper gastrointestinal bleeding from a duodenal varix, which could not be managed endoscopically; thus, the creation of an emergency TIPS was attempted. (**A**) Abdominal CT scan performed before the bleeding episode, showing the portal cavernoma. (**B**) The Colapinto needle was advanced from the right hepatic vein into a portal vein radicle using real-time sonographic and fluoroscopic guidance. (**C**) Injection of contrast shows tracking along the portal vein remnant, with stasis at the caudal aspect due to the absence of communication between the remnant and the spleno-mesenteric confluence. (**D**) A hydrophilic wire was successfully introduced through the remnant and into the spleno-mesenteric confluence. (**E**) This was substituted with a stiff wire, over which two stents were deployed in series.

**Figure 2:**
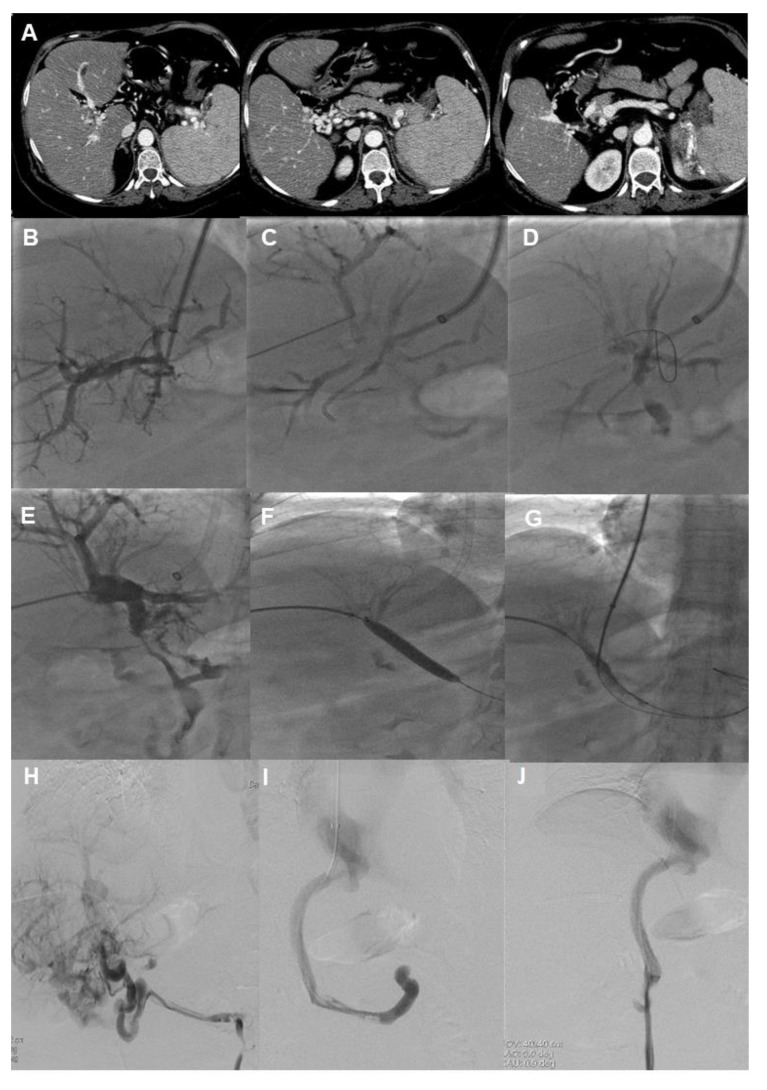
Example of a patient with idiopathic nonmalignant noncirrhotic portal vein cavernoma. The patient was admitted for recurrent upper gastrointestinal bleeding episodes from a duodenal varix, thus the creation of an elective TIPS for secondary prophylaxis of rebleeding was attempted. (**A**) Abdominal CT scan showing the portal cavernoma and patent spleno-mesenteric confluence. (**B**) The Colapinto needle was advanced from the right hepatic vein into a portal vein radicle using real-time sonographic and fluoroscopic guidance. However, it was not possible to catheterize the calcified portal vein remnant. (**C**,**D**) US-guided percutaneous puncture and subsequent catheterization of a portal vein radicle were performed with an intercostal approach. (**E**) A 6F introducer system was advanced into the intrahepatic portal vein system util the origin of the calcified portal vein remnant. (**F**) A hydrophilic wire was successfully introduced through the remnant and into the spleno-mesenteric confluence. Subsequent dilatation of the portal vein remnant was performed with 6 mm, 8 mm, and 10 mm balloon catheters. (**G**) The ballon was used as a landmark for portal vein puncture with a Colapinto needle, and subsequently, portal system catheterization was performed. (**H**) Portography performed before and (**I**) after TIPS creation with a Viatorr 10 mm diameter e-PTFE-covered stent. (**J**) Of note, there is no filling of varices at the final portography.

**Figure 3:**
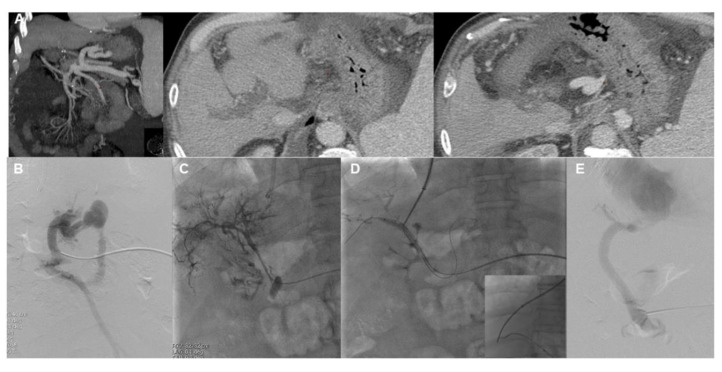
Example of a patient who developed complete intra- and extra-hepatic nonmalignant noncirrhotic portal vein thrombosis after liver transplantation. Despite receiving anticoagulant therapy, the thrombosis progressed, and the patient developed clinically significant portal hypertension, presenting with esophageal bleeding and refractory ascites. Thus, TIPS creation was pursued. (**A**) Abdominal CT scan showing complete intra- and extra-hepatic portal vein thrombosis and patent splenic vein. (**B**) The splenic vein was successfully catheterized by percutaneous US-guided puncture, and a catheter was advanced up to the spleno-mesenteric confluence. Injection of contrast shows tracking along the thrombosed portal vein. (**C**) An 8 mm balloon catheter was used for angioplasty and mechanical thrombolysis. (**D**) The ballon was then used as a landmark for portal vein puncture with a Colapinto needle, which was advanced from the right hepatic vein into a portal vein radicle, and portal system catheterization was achieved. (**E**) Portography performed after TIPS creation with a Viatorr 10 mm diameter e-PTFE-covered stent. Of note, there is no filling of varices at the final portography.

Interestingly, over the years, there has been a progressive increase in the success rate and a decrease in the short-term complication rate, which are most likely secondary to the use of covered stents, with a significant reduction in TIPS dysfunction/stenosis as compared to the use of old-generation bare metal stents, but also to improved technical skills and the integration of different percutaneous access routes. Although no high-quality prospective studies are currently available, this evidence indicates that TIPS is generally feasible in patients with chronic NNPVT and portal cavernoma, with high clinical success rates. The rate of post-TIPS hepatic encephalopathy (HE) reported in these studies ranged from 5.6% to 16.7%, while the other complications associated with TIPS creation were not adequately evaluated in all studies to draw conclusions. Survival rates after TIPS creation appear to be favorable, with most patients dying of non-liver-related causes; however, these studies were not sufficiently powered to assess survival outcomes. Although high-quality prospective studies using up-to-date materials and techniques are needed, it is likely that the only technical limitation for TIPS creation in the future, as far as NNPVT features are concerned, will be the complete absence of access to the portal system (either hepatic, splenic or mesenteric). On the other hand, diffuse biliary dilatation is still a technical contraindication to the creation of TIPS, as it can lead to the creation of bilioportal fistulas and the risk of endotypsitis in the event of biliary infection. Therefore, although no studies investigating this indication in non-cirrhotic patients with NNPVT are available to date, in cases of severe portal biliopathy with recurrent cholangitis, biliary drainage prior to TIPS/portal recanalization should be performed, as recommended for the cirrhotic population. Figure 1, Figure 2 and Figure 3 show three examples of TIPS creation in patients with portal cavernoma, showing different approaches according to the extension of the thrombus. 

In conclusion, TIPS can be applied for numerous indications in the management of NNPVT and the data are promising; however, the lack of high-quality studies and standardized protocols does not yet allow the formulation of guidelines for different indications. In general, the available data seem to confirm that the safety of TIPS use in this population is increasing over the years due to technical advancement. Moreover, liver function seems to remain intact or minimally impaired in these patients after TIPS creation, virtually eliminating the risk of liver failure and significantly reducing the occurrence of post-TIPS HE compared to patients with cirrhosis. However, these assumptions remain to be confirmed and the long-term outcomes of these patients are still unknown, so research should focus on creating high-quality studies that can answer the multiple questions posed. Moreover, it is important to emphasize that in such complex scenarios, TIPS creation should be carried out in tertiary care hospitals possessing specialized expertise in hepatology, diagnostic and interventional radiology, cardiology, abdominal surgery, and intensive care. The procedure should be conducted by physicians who are not only trained in TIPS creation but also experienced in managing potential technical complications that may arise during the procedure [51,52].

### 1.2. TIPS for Portosinusoidal Vascular Disorder

Portosinusoidal vascular disease (PSVD), previously identified as idiopathic non-cirrhotic PH, is a group of rare liver disorders of unknown aetiology [53]. PSVD histopathological diagnosis is marked by vascular irregularities that may evolve towards the onset of pre-hepatic PH in the absence of cirrhosis or any other known liver diseases causing PH [53,54]. It has been demonstrated that in these patients, the progression of PH-related complications seems to be more rapid when compared with that of patients with cirrhosis and similar liver function [55]. However, current management strategies for PH-related complications in PSVD align with cirrhosis guidelines due to the lack of specific data for this population. This includes the use of TIPS for selected patients with complications of PH, using the same indications, contraindications, and monitoring strategies as those applied to patients with cirrhosis [9,13]. The inability to accurately measure the true portosystemic gradient using the hepatic venous pressure gradient (HVPG) complicates efforts to conduct comparative studies in this population, as is conducted in cirrhosis. This limitation hinders reliable outcome predictions using HVPG, which is considered the gold standard in cirrhosis. Additionally, there is a lack of studies establishing a reference threshold to which the portosystemic gradient should be reduced following TIPS placement. Consequently, thresholds defined for cirrhosis are currently applied, although their applicability to patients with PSVD remains uncertain. In addition, the efficacy of non-selective beta-blockers is likely to be different in these patients, with a potential impact on managing secondary prophylaxis and reducing mortality rates. This underscores the need for targeted research to develop specific guidelines and treatment thresholds for PSVD patients. The lack of data specific to this population is secondary to both the rare nature of the disorder, which causes difficulties for single-center studies, and the relatively recent individuation of the disease with continuously evolving definitions and diagnostic criteria [56,57]. 

To date, the outcome of patients with PSVD treated with TIPS has been specifically described only in a few studies, which report heterogeneous results (Table 2) [58,59,60,61].

Indeed, standardizing these findings is challenging due to diverse patient profiles, procedural techniques, stent types, and insufficient clinical and technical information available in some studies. The efficacy rate varies considerably across different studies. However, the recurrence of PH-related complications appears to be closely linked to TIPS dysfunction, which was not insignificant in the studies that utilized bare stents. He and colleagues reported that TIPS proved more efficient than endoscopic therapy combined with non-selective beta blockers in preventing recurrent variceal bleeding in PSVD patients, despite there being no survival advantage noted [59]. However, the lack of comprehensive clinical details in this study makes it difficult to accurately interpret the results and compare them with those of future cohorts. Regarding TIPS-related complications, the frequency of post-TIPS HE in these patients seems similar to that reported for cirrhotic patients, ranging from 14.2% to 27%. However, predicting factors and the efficacy of treatment/prophylaxis have not yet been investigated in this population. Post-TIPS liver failure seems to be a rare event in these patients, and the incidence of post-TIPS cardiac failure has been evaluated only in one study, which reported an incidence of 2.5% (much lower than that reported for cirrhotic patients) [58]. This study, which analyzed a multicenter European cohort of 41 PSVD patients, showed excellent TIPS outcomes in patients without significant extrahepatic comorbidities and preserved renal function [58]. On the other hand, ascites as an indication for TIPS creation, the presence of portal vein thrombosis and severe comorbidities were the factors most frequently associated with poorer outcomes among different studies. Despite the existing data, the limited number of details provided in the majority of the studies and the different definitions of the disease used in different studies make it extremely difficult to draw definitive conclusions. This underscores the necessity for prospective, long-term, multicenter studies to provide more robust evidence.

## 2. Conclusions

In this review, we summarized available evidence on the use of TIPS in the non-cirrhotic population, with a focus on NNPVT and PSVD. Indeed, for these populations, there is a gap in evidence for TIPS effectiveness and safety compared to cirrhotic patients. The reported outcomes of TIPS for these diseases exhibit considerable variability, rendering comparisons challenging due to divergent study cohorts, evolving techniques across different medical centers, and shifts in practices over recent decades. Furthermore, the mixed indications for TIPS creation in the available cohorts pose a challenge in evaluating appropriate outcomes due to the variability in reporting quality among different studies. Available evidence suggests that TIPS emerges as a valuable tool for managing complications of PH and establishing portal system access in cases involving extensive NNPVT. However, the current body of evidence is limited, emphasizing the need for more comprehensive studies. To bridge this knowledge gap, it is essential to undertake high-quality long-term prospective multicenter studies to achieve sufficient numerosity considering the rarity of these conditions. These studies should offer detailed insights into the clinical characteristics of patients, outcomes, and up-to-date technical variables associated with the TIPS procedure. The latter consideration is particularly necessary for complex cases involving extensive NNPVT with cavernomatous transformation, as reported complications and technical difficulties in some studies are becoming outdated, especially in reference canters. Such an approach will not only deepen our understanding of TIPS efficacy and safety in non-cirrhotic patients but will also play a crucial role in tailoring the use of TIPS in this population, ultimately optimizing outcomes.

## Figures and Tables

**Table 1:** Safety and efficacy of TIPS creation for chronic nonmalignant noncirrhotic portal vein thrombosis with cavernomatous transformation.

Study	Study Type and Patients	Follow-Up	Indication for TIPS Creation	Access	Technical Success (%) and Details	Clinical Success	Patency	TIPS-Related Complications	Survival
Bilbao, 2004 [44]	Retrospective, observational 6 patients (100% extended to SMV, 83% to SV)	Up to 36 months (range 10–36 months)	Variceal rebleeding prophylaxis: 2 (33%) Abdominal pain: 4 (67%)	In each patient one or more approaches were tried: transhepatic (5/6), transileocolic (1/6), trans-splenic (1/6) or transjugular (1/6)	100% Type of stent: bare metal stent—in 1 patient combined with variceal embolization	Variceal rebleeding: 1 (16.7%) at 30 months	Primary patency: NA Secondary patency: 50%	NA	1 patient died during follow-up due to non-liver related cause
Senzolo, 2006 [50]	Retrospective, observational 6 patients (50% extended to SMV, 50% to SV)	NA	NA	Only transjugular access was considered	83.3% Type of stent: Memotherms (Angiomed GmbH and Co., Karlsruhe, Germany)	NA	NA	NA	NA
Fanelli, 2011 [45]	Retrospective, observational 12 patients (50% extended to SMV, 33% to SV)	Median 17.4 ± 14.7 months (range: 3.3–40.1)	Variceal rebleeding prophylaxis: 8 (66.7%) Bowel ischemia: 2 (16.7%) Need for oral anticoagulation in presence of high-risk varices: 2 (16.7%)	Only transjugular access was considered	83% Type of stent: ePTFE-covered stent ± aspiration thrombectomy	Variceal rebleeding: 1 (8.3%) which required an emergency spleno-renal shunt Recurrent intestinal ischemia: 1 at 3 and 10 months due to TIPS dysfunction, treated with mechanical thrombolysis and angioplasty or re-stenting	Primary patency: 70% Secondary patency: 90%	Transient HE: 2 (1 in the course of a severe infection) Refractory HE: 0 Liver failure: 0 patients Acute heart failure: 0	3 patients died during follow-up due to non-liver related causes
Qi, 2012 [48]	Retrospective, observational 21 patients (38% extended to SMV, 5% to SV)	Median 19.9 months (range, 3.9–96 months)	Variceal rebleeding prophylaxis: 20 (95%) Refractory ascitis: 1 (5%)	6 patients needed combined transjugular and percutaneous access	35% Type of stent: bare metal stents (8–10 mm)	Variceal rebleeding: 14%	Primary patency: 71% Secondary patency: 86%	Transient HE: 0 Refractory HE: 0 Liver failure: NA Acute heart failure: NA	2 patients died during follow-up: 1 for multiple liver abscesses 6 month after TIPS creation, and the other 1 due to non-liver related causes
Rosenqvist, 2016 [49]	Retrospective, observational 3 patients (0% extended to SMV, NA to SV)	Median, 17 months (range, 1.5–72 mo)	Variceal rebleeding prophylaxis: 3 (100%)—1 of which primary prophylaxis for variceal bleeding pre-surgery	NA	100% Type of stent: ePTFE-covered stents—in 1 patient combined with PVR	No recurrent symptoms	Primary patency: 66% Secondary patency: 100%	Overt HE: 1 patient Liver failure: NA Acute heart failure: NA	None during follow-up
Klinger, 2018 [46]	Retrospective, observational 15 patients (86.7% extended to SMV, 80% to SV)	Median 22.8 months (range 0.3–67.9 months)	Variceal rebleeding prophylaxis: 13 (76.4%) Refractory ascites: 2 (11.8%) Portal biliopathy with recurrent cholangitis: 1 (5.9%) Abdominal pain: 1 (5.9%)	Only transjugular access was considered	73.3% Type of stent: ePTFE-covered and 1 lumiex-stent (10 mm)—combined with PVR	Variceal rebleeding: 2 (11.8%) at 13 and 24 months (secondary to TIPS dysfunction for thrombus recurrence)	Primary patency: 76.5% Secondary patency: 1 and 2 year, 69.5%	Overt HE: 0 Liver failure: NA Acute heart failure: NA	3 patients died during follow-up due to sepsis (2) and intraabdominal bleeding following endoscopic retrograde cholangiopancreatography due to portal biliopathy (1)
Knight, 2021 [47]	Retrospective, observational 39 patients (74.4% extended to SMV, 71.7% to SV)	Up to 72 months	Variceal rebleeding prophylaxis: 24 (61.5%) Ascites 6: (15.4%) Abdominal pain: 23 (59.0%) Bowel ischemia: 1 (2.6%) Portal cholangiopathy: 1 (2.6%)	20 patients (69.2%) needed percutaneous access	100% Type of stent: NA	1 presented with minor variceal rebleeding after TIPS due to stent dysfunction, which was corrected	At 36 months, 63% free of primary TIPS thrombosis; 81% when incorporating additional management of TIPS (angioplasty, re-stenting)	Transient HE: 2 (5.1%) Refractory HE: 1 (2.6%), treated with TIPS recalibration Liver failure: 0 Acute heart failure: 1 (2.6%), managed with diuresis	NA

Legend: TIPS = transjugular portosystemic shunt; SMV = superior mesenteric vein; SV = splenic vein; HE = hepatic encefalopahty; PVR = percutaneous venous recanalization; NA = not available.

**Table 2:** Safety and efficacy of TIPS creation for portosinusoidal vascular disorder.

Study	Study Type and Patients	Follow-Up	Indication for TIPS Creation	Associated Conditions	Technical Success (%) and Details	Clinical Success	Patency	TIPS-Related Complications	Survival	Notes
Bissonnette, 2016 [58]	Retrospective, observational 41 patients, between 2000–2014 Associated portal vein thrombosis: 16 (39%including 3 cavernomas)	Mean 27 ± 28 months	Variceal bleeding: 25 (61%) urgent: 19 (76%) pre-emptive 6 (24%) Refractory ascites: 16 (39%)	Idiopathic: 34% HIV: 10% Immunological disorders: 22% Exposure to toxic agents/neoplasia: 17% Prior transplantation: 15% Prothrombotic states: 5%	100% Type of stent: ePTFE-covered stent in 80%, bare metal stent TIPS in 20% PPG: 19 ± 6 mmHg to 7 ± 3 mmHg	Variceal rebleeding: 7 (28%)—Early stent thrombosis accounted for 3, which were successfully managed with TIPS revision. Ascites persistence/recurrence: 6 (33%)—All controlled with low-dose diuretic	Primary patency: 73% Secondary patency: 100%	Transient HE: 11 (27%) Refractory HE: 2 (5%)—treated by shunt reduction Liver failure: none Acute heart failure: 1 (2.5%)	During follow-up, 11 patients died (27%): 5 intrahospital deaths	Pre-TIPS creatinine and splanchnic vein thrombosis were associated with post-TIPS HE risk. Creatinine, ascites as indication for TIPS, and associated comorbidities were associated with mortality risk post-TIPS.
Regnault, 2018 [61]	Retrospective, observational 25 patients, between 2004–2015 (5 were not PSVD) Associated portal vein thrombosis: 5 (20%—including 3 cavernomas)	Mean 39 ± 37 months	Variceal rebleeding prophylaxis: 14 (56%) Ascites: 5 (20%) Variceal rebleeding prophylaxis + ascites: 5 (20%) Pre-surgical: 1 (4%)	Idiopathic: 16% Exposure to toxic agents/neoplasia: 20% Prothrombotic states: 28% Others: 16% Non-PSVD: 20%	100% Type of stent: ePTFE-covered TIPS in 88%, bare metal stent TIPS in 12% + variceal embolization in 10 cases, and partial splenic embolization in 3 PPG: 14.7 ± 2.8 mmHg to 5 ± 2.3 mmHg	Variceal rebleeding: 4% Ascites persistence/recurrence: 12%	Primary patency: 80% Secondary patency: 100%	Transient HE: 5 (20%) Refractory HE: 5 (20%)—3 treated with TIPS recalibration and 2 did not resolve Liver failure: 1 (4%) Acute heart failure: NA	During follow-up, 6 patients died (24%): 1 misposition of a covered stent 2 recurrence of PH complications (1 after early stent thrombosis and 1 after TIPS recalibration for HE) 3 non-liver related deaths	Two out of three patients with cavernoma and all excluded for insufficient data but similar anatomy faced early complications or failures. Of the surviving nine, three had ascites recurrences, which significantly linked to mortality.
Lv, 2019 [60]	Retrospective, observational 76 patients, between 2001 and 2015 Associated portal vein thrombosis: 29 (38%—including 3 cavernomas)	Median 36.4 months (IQR 23.0–62.5)	Variceal rebleeding prophylaxis: 66 (86.8%) Emergency TIPS: 10 (13.2%)	Immunological disorders: 11.8% Exposure to toxic agents/neoplasia: 2.6% Prothrombotic states: at least one pro- thrombotic disorder was present in 32.9%	100% Type of stent: ePTFE-covered stent in 78%, bare metal stent TIPS in 22% PPG: 25.5 ± 4.7 mmHg to 8.8 ± 3.5 mmHg	Variceal rebleeding: 33% Ascites persistence/recurrence: 12%	Primary patency: NA Secondary patency at 5 years: 65%	Overt HE: 11 (14%) Liver failure: 1 (4%) Acute heart failure: NA	During follow-up, 9 patients died (12%): patients with The 1-, 2- and 5-year actuarial mortality probabilities were 4%, 7% and 11%	Severe associated disorders and ascites were associated with a higher mortality rate
He, 2020 [59]	Retrospective, observational 28 patients, between 2012 and 2015	Up to 3 years	Variceal rebleeding prophylaxis: 100%	NA	100% Type of stent: ePTFE-covered stent in + embolization of coronary gastric vein PPG: 29.2 ± 6.1 mmHg to 9.7 ± 5.2 mmHg	Variceal rebleeding: 7.1%	Shunt stenosis was found in 4 patients (14.2%)	Overt HE: 4 (14.2%) Liver failure: NA Acute heart failure: NA	Accumulated mortality was 3.6%	This study compared different treatments for controlling variceal bleeding in patients with PSVD. TIPS and esophagogastric devascularization were superior to endoscopic therapy + non-selective β-blockers for secondary prevention of variceal bleeding but not in improving survival.

Legend: TIPS = transjugular portosystemic shunt; PPG = portal pressure gradient; HE = hepatic encefalopahty; PSVD = portosinusoidal vascular disorder; PH = portal hypertension; IQR = interquartile range; NA = not available.

## Abbreviations

Transjugular intrahepatic portosystemic shunt (TIPS); portal hypertension (PH); nonmalignant noncirrhotic portal vein thrombosis (NNPVT); portosinusoidal vascular disorder (PSVD); superior mesenteric vein (SMV); splenic vein (SV); portal vein recanalization (PVR); hepatic encephalopathy (HE); hepatic venous pressure gradient (HVPG).
